# Treating skin involvement in diffuse cutaneous systemic sclerosis: results from an international scleroderma specialist survey

**DOI:** 10.1093/rap/rkag086

**Published:** 2026-07-21

**Authors:** Kimberly S Lakin, Antonia Valenzuela, John D Pauling, Deanna P Jannat-Khah, Kelsey Gripp, Oshmita Golam, Robyn T Domsic, Robert F Spiera, Jessica K Gordon

**Affiliations:** Department of Medicine, Hospital for Special Surgery, New York, NY, USA; Department of Medicine, Weill Cornell Medicine, New York, NY, USA; Department of Medicine, Pontificia Universidad Católica de Chile, Santiago, Chile; Department of Clinical Immunology and Rheumatology, UC CHRISTUS Healthcare System, Santiago, Chile; Department of Medicine, North Bristol NHS Trust, Bristol, UK; Department of Medicine, Hospital for Special Surgery, New York, NY, USA; Department of Medicine, Weill Cornell Medicine, New York, NY, USA; Department of Medicine, Hospital for Special Surgery, New York, NY, USA; Department of Medicine, Hospital for Special Surgery, New York, NY, USA; Division of Rheumatology & Clinical Immunology, Department of Medicine, University of Pittsburgh, Pittsburgh, PA, USA; Department of Medicine, Hospital for Special Surgery, New York, NY, USA; Department of Medicine, Weill Cornell Medicine, New York, NY, USA; Department of Medicine, Hospital for Special Surgery, New York, NY, USA; Department of Medicine, Weill Cornell Medicine, New York, NY, USA

**Keywords:** systemic sclerosis, scleroderma, diffuse cutaneous systemic sclerosis, skin fibrosis, real-world practice, treatment guidelines, physician survey, immunosuppressive therapies, biologic therapy

## Abstract

**Objectives:**

To evaluate contemporary global treatment of diffuse cutaneous systemic sclerosis (dcSSc) skin by SSc specialists.

**Methods:**

An anonymous survey was distributed via the Scleroderma Clinical Trials Consortium, European Scleroderma Trials and Research Group (EUSTAR), and Collaborative National Quality and Efficacy Registry (CONQUER) distribution lists between June and September 2025. The survey comprised four sections: Demographics, Treatment of dcSSc Skin, Impact of Recent Guidelines, and Clinical Trials.

**Results:**

Responses included 103 physicians (93 completed): 43% North America, 31% Europe, 15% South America and 12% elsewhere. The majority practice within an SSc centre (80%) and participate in clinical trials (84%). Mycophenolate mofetil (MMF) was preferred first-line treatment (71%) for dcSSc without ILD, rising to 92% for dcSSc with mild/non-progressive ILD. For active skin involvement despite MMF, trial referral was preferred as next step irrespective of ILD (59% without ILD and 58% with ILD). Treatment preferences have evolved, with 40% increasing MMF use and 47% decreasing methotrexate use, over the previous 2–3 years. Biologic use has increased, with 56% and 37% reporting increasing rituximab and tocilizumab use, respectively. For dcSSc without ILD, 85% have prescribed rituximab, and 65% have prescribed tocilizumab. Biologic availability has impacted trial enrolment (73% agreement). Treatment decision-making for dcSSc skin involvement has been materially influenced by recent BSR and/or EULAR guidelines (43% agreement).

**Conclusion:**

MMF is preferred first-line for dcSSc. In the absence of compelling scientific justification for combination or step-up immunomodulatory approaches, trial referral is currently the preferred next treatment option. Biologic use is increasing, which impacts trial enrolment.

Key messages

- MMF is the preferred first-line agent for managing skin involvement in diffuse cutaneous systemic sclerosis (dcSSc) irrespective of ILD in contemporary, real-world practice.
- Biologic use in dcSSc is increasing and may impact clinical trial enrolment.
- Treatment decision-making has been materially influenced by recent clinical practice guidelines in almost half of SSc specialists.

## Introduction

While cardiopulmonary disease is the leading cause of death in systemic sclerosis (SSc), diffuse skin involvement causes significant morbidity related to pain, itch and functional impairment [1–3]. Treatment options that fully address both cutaneous and internal organ manifestations are needed, underscoring the continued importance of SSc clinical trials. Fortunately, dcSSc treatment options are expanding and international guidelines are evolving, underscoring the dynamic landscape of SSc skin treatment as well as the need for an updated assessment of the contemporary, real-world, standard-of-care for managing diffuse skin fibrosis.

Historically, methotrexate was the preferred first-line treatment for diffuse skin treatment per 2012 [4] and 2018 [5] SSc specialist surveys and was the only treatment endorsed in the 2017 European Alliance of Associations for Rheumatology (EULAR) SSc recommendations for managing skin fibrosis [6]. However, clinical data have since accrued regarding the use of mycophenolate mofetil (MMF) [7], rituximab [8, 9], and tocilizumab [10] for dcSSc. Accordingly, the European Alliance of Associations for Rheumatology (EULAR) as well as the British Society for Rheumatology (BSR) released updated SSc management guidelines in 2024, which included recommendations for treating diffuse skin fibrosis. Compared with the 2017 guideline, EULAR newly added mycophenolate mofetil (MMF) and rituximab, in addition to methotrexate, as options for dcSSc skin [11]. Tocilizumab is also recognized as an additional option for treating skin involvement among those with early, inflammatory dcSSc, citing a trend toward benefit and a favourable safety profile, despite the fact that the phase III clinical trial did not meet its primary end point for skin improvement [10]. The BSR guideline recommends MMF for early dcSSc with methotrexate as an alternative [12]. It is unknown what influence these guidelines are having on SSc treatment patterns and whether these two European-based guidelines reflect global physician practices for managing diffuse skin fibrosis. It is timely and essential to characterize the global, contemporary standard-of-care for managing diffuse SSc skin fibrosis to contextualize the guidelines as well as to inform clinical trials designed to compare novel treatments to standard-of-care.

The purpose of this study is to survey SSc specialists to characterize the contemporary standard-of-care for treating dcSSc skin involvement, with a focus upon biologic use, guideline alignment and influence and referral patterns for clinical trials.

## Methods

An anonymous survey regarding treatment of dcSSc skin involvement was sent to the email distribution lists for the Scleroderma Clinical Trials Consortium (SCTC), European Scleroderma Trials and Research Group (EUSTAR) investigators, and Collaborative National Quality and Efficacy Registry (CONQUER) investigators by the respective organizations on our behalf. Ten days following the initial invitation, a reminder email was distributed to the SCTC. The SCTC is an international organization of healthcare professionals with expertise in SSc care and research and includes 116 institutions across 29 countries within North America, South America, Europe, Asia, and Australia, with approximately 232 active members at the time of survey distribution [13]. EUSTAR is an international consortium that included 145 active centres worldwide at the time of survey distribution, across 47 countries and six continents, with one to two investigators at each site [14]. CONQUER investigators are US-based physicians with expertise in SSc clinical care and research [15]. This survey was sent to 27 CONQUER investigators across 19 sites. Because the survey was sent anonymously to organizational email distribution lists on our behalf and physicians could be members of more than one organization, accurate response rates could not be calculated; characteristics of responders and non-responders could not be compared, and failure to deliver messages was not quantified.

The survey was administered through REDCap and available between June and September 2025 and contained four sections: Demographics, Treatment of dcSSc Skin Involvement, Impact of Recent Guidelines, and Clinical Trials for dcSSc (Supplemental Material, available at *Rheumatology Advances in Practice* Online). Each of these sections was delivered on a separate form that required advancement. An answer to each question was required to advance. Branching logic was used to optimize survey efficiency and user experience. In the Recent Guidelines section, a summary of the BSR and EULAR guidelines for managing diffuse skin involvement was provided. ‘Please Specify’ responses were manually reviewed. For first- and second-line treatment questions, ‘Other’ responses were reviewed and, if clearly assignable to an existing response, were reclassified, as appropriate. Other responses were reviewed, standardized for format and spellin, and included in the Supplemental Material, available at *Rheumatology Advances in Practice* Online.

A descriptive statistical analysis was used to analyse the survey data. Response frequencies were summarized as counts and percentages. First-line treatment preferences were described in the full cohort and by region. No stratified sampling or post-stratification weighting was performed. Microsoft Excel (version 2604) and STATA version 18.0 were used to generate figures and perform tabulations.

Ethics approval was obtained from the Hospital for Special Surgery Institutional Review Board (Study Number 2025-1176) with a waiver of documentation of informed consent due to the anonymous study design. Participants were informed that completing and submitting the questionnaire represented consent to participate in a research study, in accordance with the Declaration of Helsinki.

## Results

### Respondent characteristics

One hundred and three physicians responded, with 93 (90%) completing the full survey. Clinical practice and demographics were generally similar between completers and non-completers (Supplementary Table S1, available at *Rheumatology Advances in Practice* Online). The estimated response rate is 19–44%, as the exact number of active members in each organization and the number of individuals with dual memberships are unknown due to the anonymous study design. Respondents mostly practiced at academic institutions (94%) with dedicated SSc centres (80%) and had prior experience participating in SSc clinical trial(s) (84%), with 30% reporting that they see >50 patients with SSc (of any subtype) per month (Table 1). Geographically, the most common practice locations were North America (43%), Europe (31%) and South America (15%). Respondents were well distributed with respect to years in practice (≤10 years: 34%, 11–20 years: 26%, >20 years: 40%).

**Table 1:** Characteristics of 103 physician respondents.

Characteristic	*N* = 103
Sex	
Female	62 (60%)
Male	39 (38%)
Prefer not to answer	2 (2%)
Age group, years	
20–40	24 (23%)
41–60	51 (50%)
61–80	28 (27%)
Region	
North America	44 (43%)
Europe	32 (31%)
South America	15 (15%)
Australia and Oceania	5 (5%)
Middle East and North Africa	2 (2%)
Southeast Asia	3 (3%)
South Asia	1 (1%)
East Asia	1 (1%)
Years in practice	
≤10 years	35 (34%)
11–20 years	27 (26%)
>20 years	41 (40%)
Practice at an academic center	97 (94%)
Practice at a dedicated systemic sclerosis (SSc) center	82 (80%)
Prior experience as principal or co-investigator for SSc trial(s)	87 (84%)
Age group of patients in practice	
Adults only	93 (90%)
Adults and children	10 (10%)
Monthly SSc patient volume	
≤20 patients	37 (36%)
21–50 patients	35 (34%)
>50 patients	31 (30%)

### First- and second-line therapies for dcSSc skin involvement

**Figure 1:**
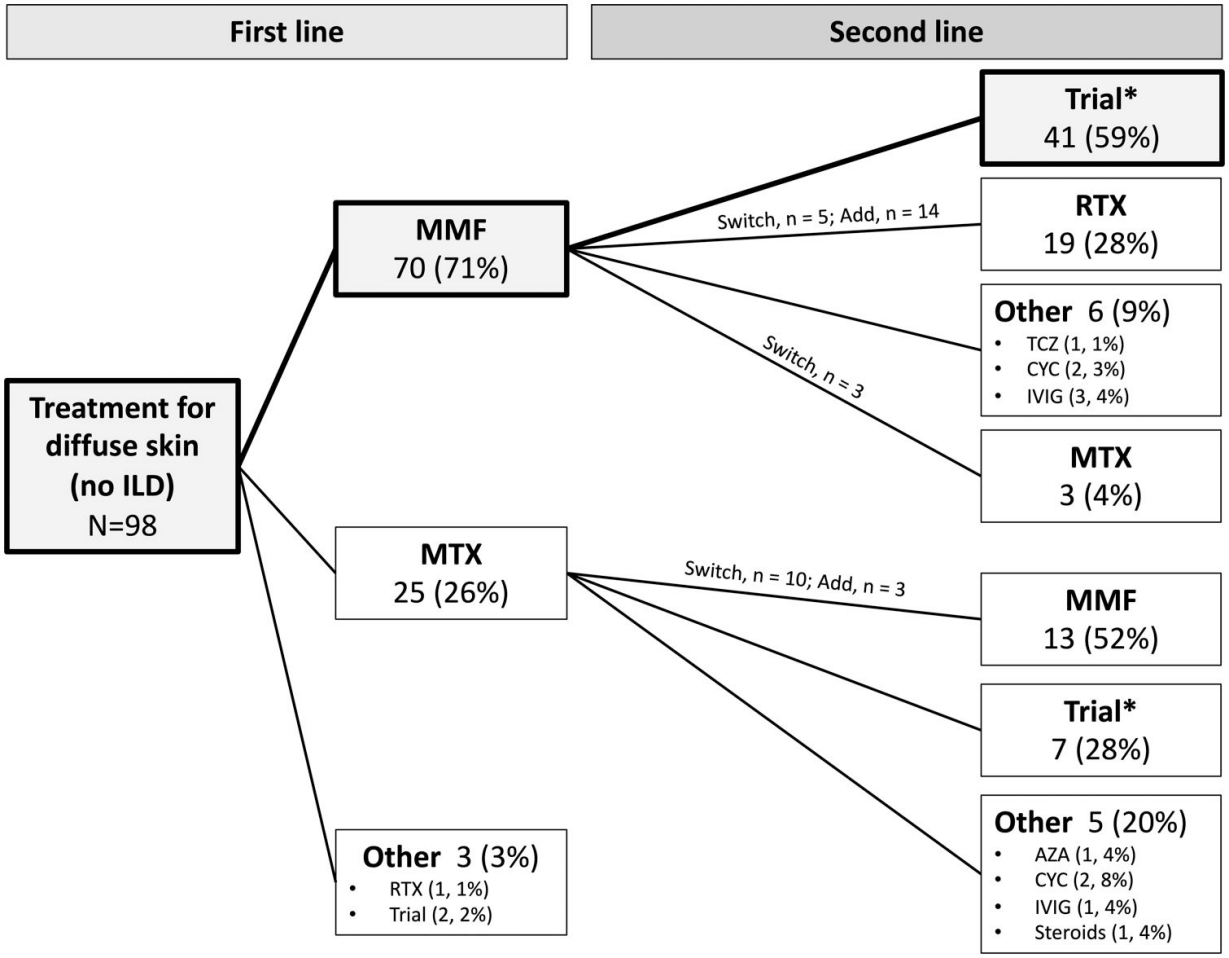
Initial treatment approach to managing diffuse skin involvement in systemic sclerosis for patients without interstitial lung disease. *Trial referral as a second-line approach after mycophenolate included 10 respondents who would refer and add another agent (8 rituximab, 1 methotrexate, 1 intravenous immunoglobulin). Trial after methotrexate included 1 respondent who would refer and add rituximab and 3 who would refer and switch to mycophenolate. For one respondent, while MMF was a first-line option, the second-line treatment preference depended on clinical severity. ILD, interstitial lung disease; MMF, mycophenolate mofetil or mycophenolic acid; MTX, methotrexate; RTX, rituximab; TCZ, tocilizumab; CYC, cyclophosphamide; AZA, azathioprine; IVIG, intravenous immunoglobulin

Ninety-eight respondents completed the survey questions related to initial approaches to diffuse skin treatment. For patients with dcSSc without ILD, the preferred first-line treatment for skin involvement was MMF (*n* = 70, 71%), while 25 respondents (26%) preferred methotrexate (Fig. 1). Among those who preferred MMF as first-line, if it was not effective, 41 respondents (59%) would refer to a clinical trial and 19 (28%) would prescribe rituximab (5 would switch to rituximab, 14 would add rituximab). Among those who preferred methotrexate as first line, if it was not effective, 13 respondents (52%) would prescribe MMF next (10 would switch to MMF, 3 would add MMF), and 7 (28%) would refer to a clinical trial.

**Figure 2:**
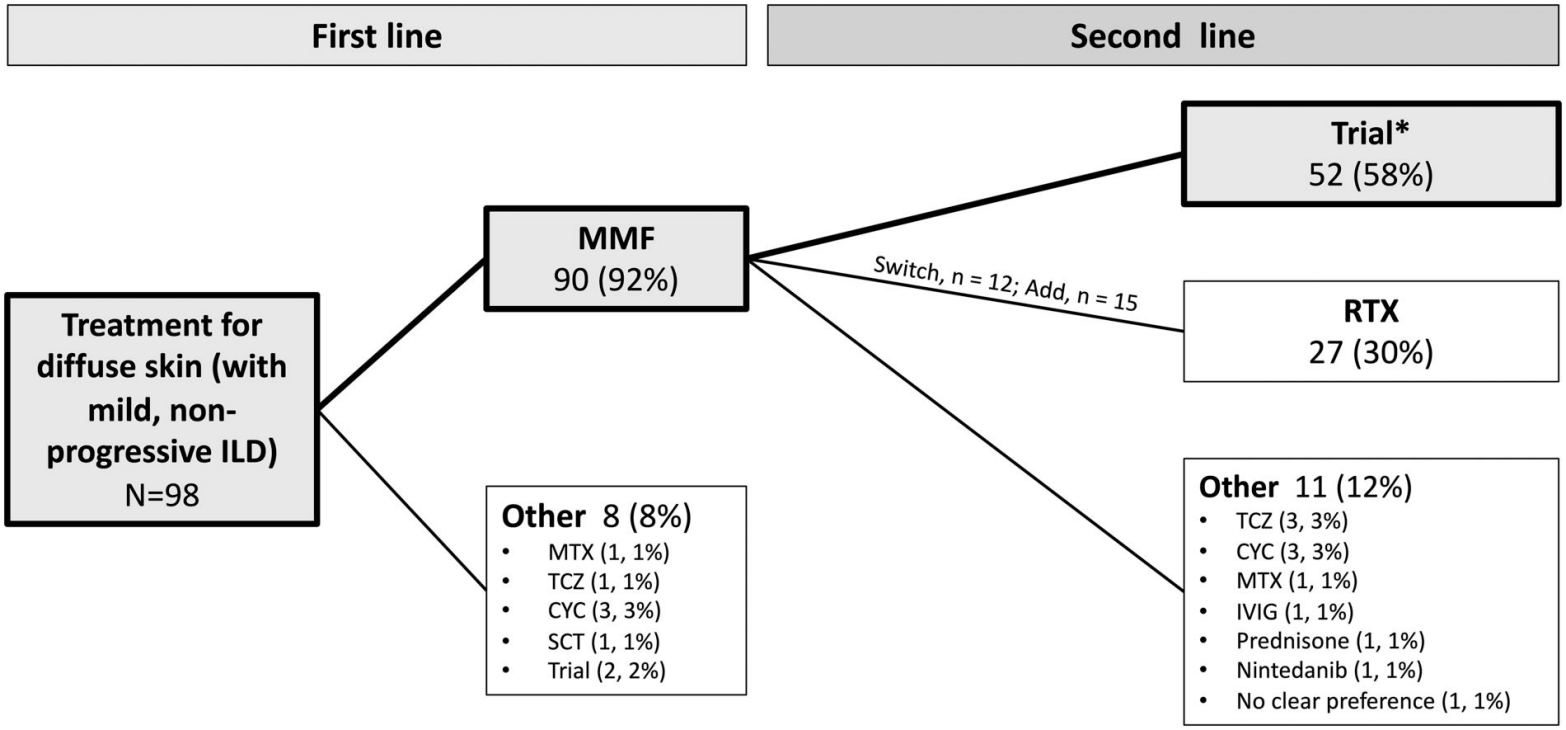
Initial treatment approach to managing diffuse skin involvement in systemic sclerosis for patients with mild/non-progressive interstitial lung disease. *Trial referral includes 12 respondents who refer patients to a clinical trial and add rituximab, 2 who refer and switch to rituximab, 1 who refers and adds methotrexate, 3 who refer and add tocilizumab, and 3 who refer and switch to tocilizumab. The remainder refer and continue mycophenolate. ILD, interstitial lung disease; MMF, mycophenolate mofetil or mycophenolic acid; MTX, methotrexate; RTX, rituximab; TCZ, tocilizumab; CYC, cyclophosphamide; SCT, stem cell transplant; IVIG, intravenous immunoglobulin

For patients with dcSSc with mild, non-progressive interstitial lung disease, MMF was preferred as first line by 90 respondents (92%) (Fig. 2). Among those who prefer MMF first line, if it was not effective, 52 (58%) would refer to a clinical trial, and 27 (30%) would prescribe rituximab (12 would switch to rituximab, and 15 would add rituximab).

**Figure 3:**
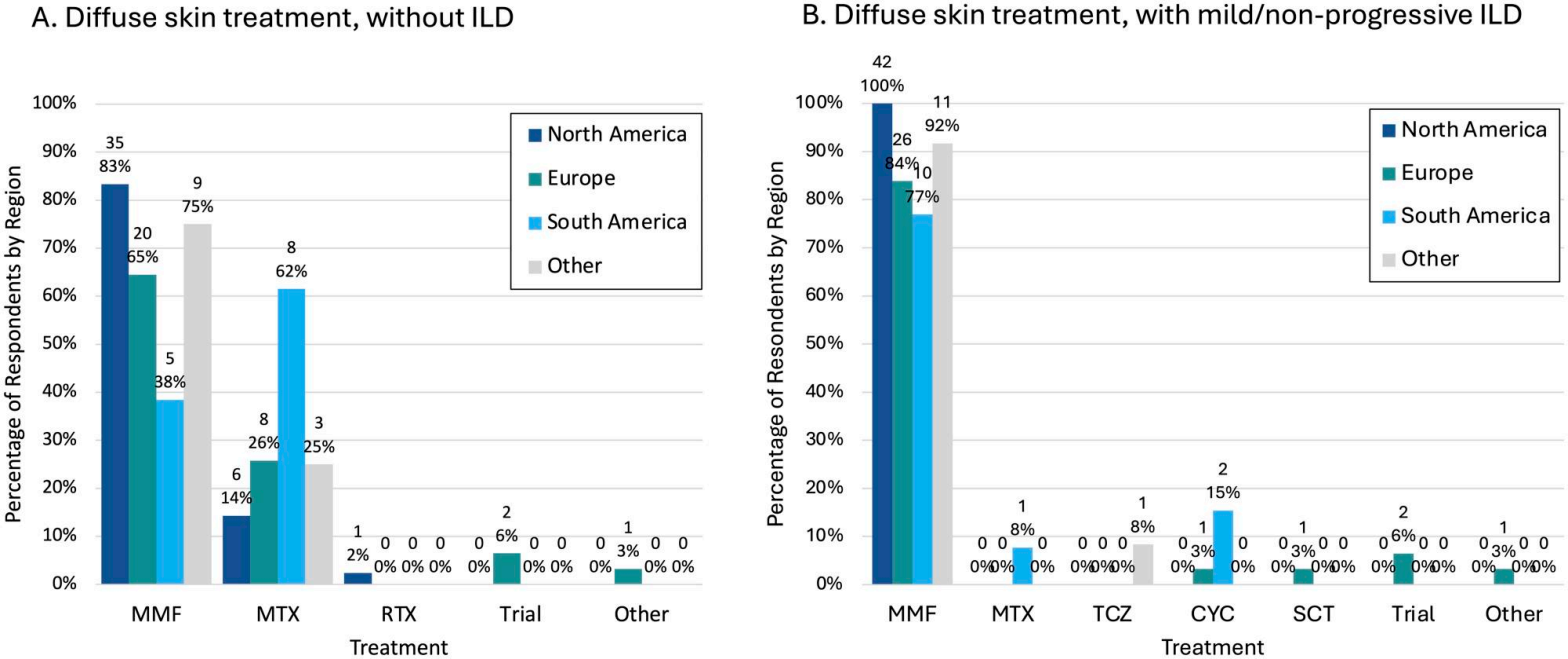
Preferred first-line treatment option for diffuse skin involvement in systemic sclerosis in patients (A) without interstitial lung disease and (B) with mild/non-progressive interstitial lung disease, stratified by region. ILD, interstitial lung disease; MMF, mycophenolate mofetil or mycophenolic acid; MTX, methotrexate; RTX, rituximab; TCZ, tocilizumab; SCT, stem cell transplant

First-line preferences were evaluated by region for the management of diffuse skin involvement in the absence of ILD and in the presence of mild, non-progressive ILD (Fig. 3). North American respondents had the highest preference for MMF across regions, with 100% indicating they prefer MMF first line in cases of ILD. In South America, overall preference for MMF or methotrexate as first-line treatment for skin depended on the patient’s pulmonary status. Specifically, methotrexate was preferred first line by 62% of respondents when there is no ILD, but MMF was preferred first line by 77% when mild/non-progressive ILD is present.

Most respondents indicated that they determine the effectiveness of their first-line therapy in 6 months or sooner (3 months: 40%; 6 months: 43%) (Supplementary Fig. S1, available at *Rheumatology Advances in Practice* Online). Only 1% indicated they require 12 months to determine whether a first-line medication was successful.

**Figure 4:**
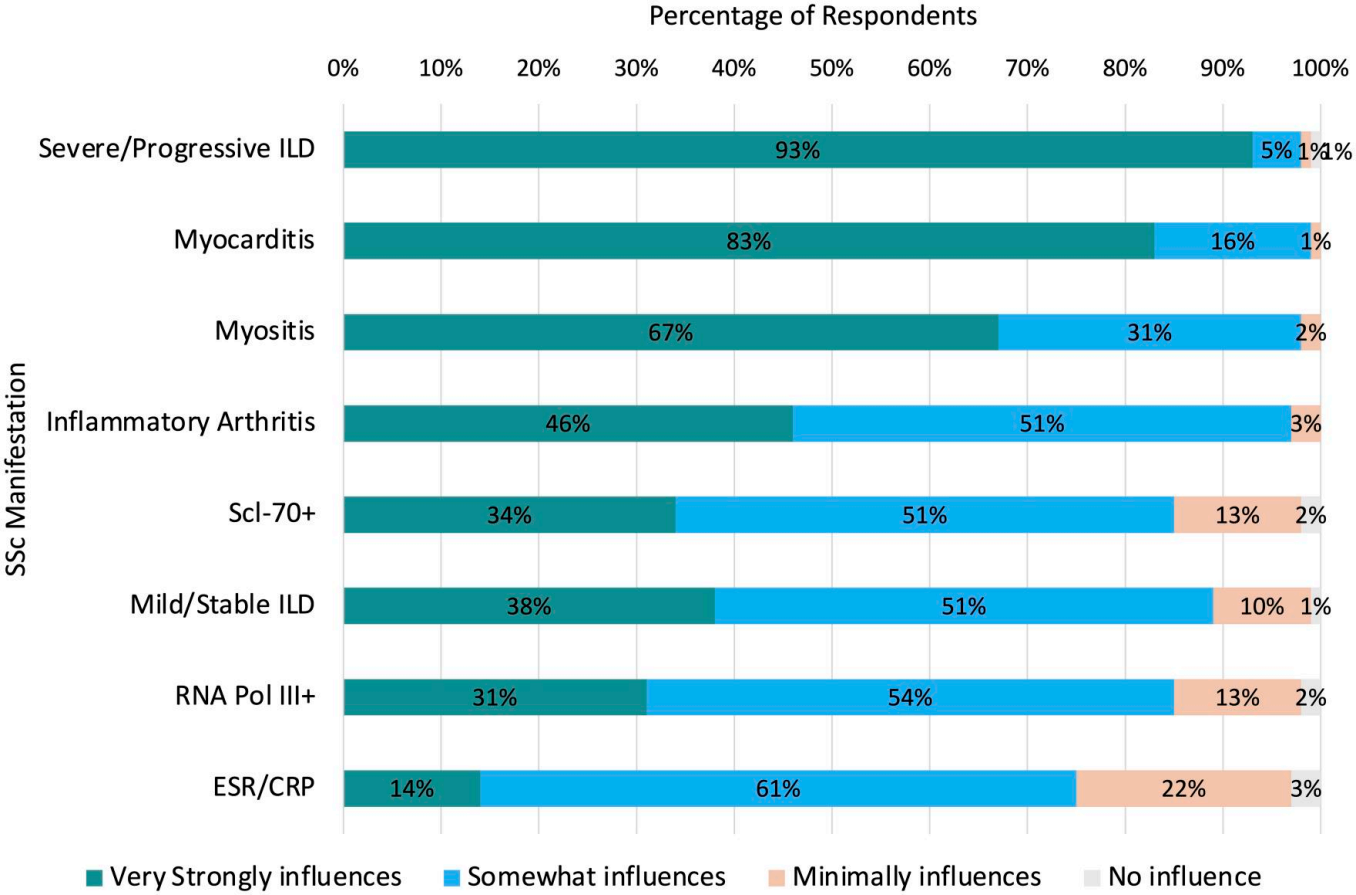
Influence of other systemic sclerosis manifestations on the preferred initial treatment for diffuse skin involvement. ILD, interstitial lung disease; RNA Pol III, RNA polymerase III autoantibody; ESR, erythrocyte sedimentation rate; CRP, C-reactive protein

As a systemic disease, diffuse skin involvement generally does not occur in isolation, and respondents reported that other SSc clinical features influence their decision regarding the selection of first-line treatment for skin (Fig. 4). The top features that respondents reported ‘very strongly influences’ first-line diffuse skin treatment decisions were severe/progressive ILD (93%), myocarditis (83%), myositis (67%), inflammatory arthritis (46%), and Scl-70 autoantibody positivity (34%).

For patients with active skin disease as well as myocarditis, most respondents preferred to use upfront combination therapy (31 of 98, 32%), followed by MMF monotherapy (*n* = 26, 27%), and cyclophosphamide (*n* = 21, 21%). Of those 31 respondents who prefer upfront combination therapy, 17 (55%) preferred combining MMF with rituximab.

Respondents were also asked their preferred approach to managing diffuse skin involvement for a patient planning pregnancy. For managing significant skin disease without heart or lung involvement in the setting of desired pregnancy for a patient taking MMF or methotrexate, the most common preferred action was to switch MMF/methotrexate to azathioprine (38%), while 16% stop MMF/methotrexate and monitor off therapy, and 15% switch MMF/methotrexate to rituximab (see Supplementary Table S2, available at *Rheumatology Advances in Practice* Online for summary of all potential responses).

### Biologics

**Figure 5:**
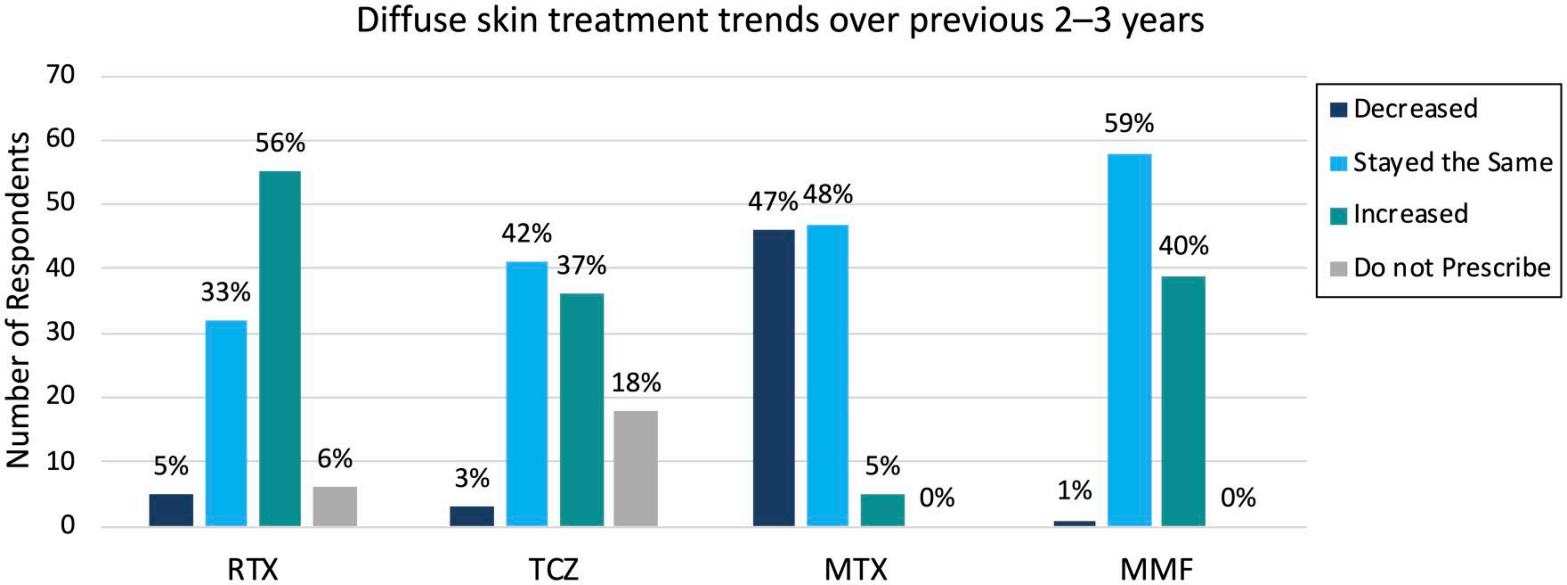
Treatment trends over the previous 2–3 years for managing diffuse cutaneous systemic sclerosis skin involvement. RTX, rituximab; TCZ, tocilizumab; MTX, methotrexate; MMF, mycophenolate mofetil or mycophenolic acid

All respondents reported that they were able to access rituximab for treating diffuse skin involvement (easy access: 66%; limited/difficult access: 34%; no access: 0%). Regional distribution regarding rituximab access is shown in Supplementary Table S3, available at *Rheumatology Advances in Practice* Online. 85% have used rituximab for diffuse skin involvement specifically. Over the last 2–3 years, 56% reported an increase in their prescribing of rituximab for skin (Fig. 5).

Most respondents reported that they were able to access tocilizumab for treating diffuse skin involvement (easy access: 63%; limited/difficult access: 34%; no access: 3%), and 65% have used it for managing diffuse skin involvement among individuals without ILD. Limited access or no access to tocilizumab was more common among 15 respondents from South America, compared with other regions (Supplementary Table S3, available at *Rheumatology Advances in Practice* Online). While 29% of respondents felt that tocilizumab was not helpful for skin or that more data was needed, 18% would prescribe in cases of refractory skin involvement, and 41% would prescribe tocilizumab for skin involvement, only if inflammatory arthritis was also present. Tocilizumab use for treating diffuse skin involvement is increasing, with 37% of respondents indicating that they have prescribed it more often over the last 2–3 years (Fig. 5).

### Autologous hematopoietic stem cell transplant (HSCT)

Stem cell transplant programs are available at the practice location of 39% of respondents. Most respondents refer for HSCT only in cases of refractory skin involvement (68%), while 6% refer any eligible patient, with similar distributions among North American and European respondents (North America: 78%; Europe: 77%; South America: 69%; Other: 55%). Of those who do not refer for HSCT, 15% do not have access, 7% feel that HSCT is not more effective than other available therapies, and 4% feel that the risks of HSCT outweigh the benefits.

### Corticosteroid use

While endorsed by neither the BSR nor EULAR guidelines, most respondents reported using corticosteroids for diffuse skin involvement; however, the use and dose depend on the patient’s RNA polymerase III autoantibody status (Supplementary Fig. S2, available at *Rheumatology Advances in Practice* Online). For RNA polymerase III-positive patients, 42% of respondents avoid any dose of corticosteroids when treating skin, while 27% would avoid corticosteroids in RNA polymerase III-negative patients. For those who prescribe corticosteroids for skin, the most common maximum dose was 10 mg of prednisone equivalent.

### Treatment of non-fibrotic skin manifestations

Respondents were asked to indicate if they have prescribed or would prescribe various treatment options for itch in dcSSc. The most common treatment for itch used by respondents was antihistamines (84%), followed by gabapentin/pregabalin (61%), low-dose oral corticosteroids (49%), topical steroids (37%), low-dose naltrexone (30%) and mirtazapine (20%). For telangiectasias, skin camouflage (such as makeup with green tint) was recommended by 74%. Only a slight majority (58%) recommend laser therapies such as pulsed dye laser (PDL) and intense pulsed light (IPL) for telangiectasia. While the BSR recommendations mention, without directly recommending, the use of injected sclerosing agents for telangiectasias, only 2% of respondents have or would prescribe this intervention.

### Guideline alignment

Respondents were familiar with recent guidelines, with 95% and 85% reporting having either heard a presentation about and/or read the EULAR and BSR guidelines, respectively. About 75% described the EULAR guideline as fully or mostly reflecting their current approach for managing diffuse skin fibrosis, and 60% reported that the BSR recommendations fully or mostly reflect their current treatment approach (Supplementary Fig. S3, available at *Rheumatology Advances in Practice* Online). Of 94 respondents, 40 (43%) indicated that they have or will make changes to their therapeutic approach to dcSSc based upon the recent BSR and/or EULAR guidelines.

### Clinical trials

Seventy-three percent of respondents agreed that biologics have impacted clinical trial enrolment. For patients who failed MMF, 33% prefer to prescribe RTX rather than refer to a trial for those without ILD (31% neutral), and 44% prefer to prescribe RTX rather than refer to a trial for those with ILD (18% neutral). Only 14% of respondents were willing to enrol patients with early, active dcSSc in a 12-month trial without background therapy. A higher proportion of respondents, though still a minority, would consider enrolling patients into placebo-controlled 6-month trials without background therapy (26% would enrol patients without ILD; 13% would enrol patients with ILD). However, if the protocol had a clear escape strategy, 66% would enrol patients with dcSSc in a placebo-controlled trial without background therapy. Although 72% were comfortable with uniform background MMF, 82% preferred background therapy to be at their discretion. Skin biopsies are often collected in clinical trials, and 53% felt that these procedures did not represent a significant barrier for trial enrolment (31% neutral).

All ‘other, please specify’ responses are summarized and shown in Supplementary Table S4, available at *Rheumatology Advances in Practice* Online.

## Discussion

The current treatment landscape for dcSSc skin involvement according to SSc specialists has shifted away from methotrexate and toward MMF and biologic therapies, highlighting the impact of recent clinical studies and guidelines.

The preference for MMF first-line for dcSSc skin involvement contrasts with prior studies, reflecting both emerging clinical data, including post-hoc analyses of the lenabasum and Scleroderma Lung Study-I and -II trials [7, 16], as well as guideline evolution [6, 11, 12]. In 2012, a survey of 78 members of the SCTC and/or Canadian Scleroderma Research Group reported that methotrexate was the most preferred first-line treatment for SSc skin involvement, regardless of skin severity [modified Rodnan skin score of 10 (mild), 24 (moderate) or 32 (severe)], with 77% indicating that they would add MMF to methotrexate as second line [4]. Among those who use methotrexate second line, respondents in our study now preferred to switch rather than add, contrasting these results. Subsequently, in 2018, SSc specialists preferred methotrexate for mild and moderate skin involvement, but MMF for cases of severe skin involvement, using the same mRSS thresholds of skin severity [5], which were not used in our study. These two international studies aligned with the 2017 EULAR SSc treatment recommendations stating that methotrexate may be considered for skin fibrosis, and that MMF had not yet been extensively studied for skin [6]. Our study demonstrates a strong preference for MMF for dcSSc skin regardless of ILD status and aligns with the 2024 guidelines [11, 12].

In our study, there was regional variation with respect to the preferred first-line treatment for dcSSc skin: North American respondents had a strong preference for MMF regardless of ILD presence, while South American respondents preferred methotrexate for patients without ILD. A 2025 study reported that methotrexate was also the preferred first-line treatment for dcSSc skin in India [17]. These regional differences may reflect cost, availability, local public formularies, methotrexate familiarity, as well as geographical variation in clinical approaches to SSc.

Clinical trials were preferred as second-line treatment of dcSSc skin, which contrasts prior literature that positioned clinical trials as a later-line consideration. Specifically, in the 2018 SSc specialist survey, trials were fifth line, preferred for severe dcSSc skin involvement, and considered after MMF, methotrexate, cyclophosphamide and stem cell transplantation; 71% of 2018 respondents agreed with this algorithm [5]. The second-line referral to clinical trials in our study may be due to the inclusion of early disease in trial enrolment criteria, the enthusiasm regarding the mechanisms of action of contemporary studies, and the high participation of respondents in SSc trials. The absence of compelling scientific justification for combination or step-up immunomodulatory approaches to early dcSSc management may also be an important driver for considering trial referral as the preferred next treatment option following MMF. Of note, most respondents would not enrol patients in 6- or 12-month placebo-controlled trials without background therapy, reflecting a shift compared with some recent trial designs [8, 10]. Respondents also preferred that background therapy be at their discretion, rather than determined by the trial protocol. These data may be used to inform future SSc clinical trial designs to enhance feasibility and recruitment.

The role of biologics such as rituximab and tocilizumab for managing dcSSc skin is increasing and has influenced referral to clinical trials. The tocilizumab phase III study comparing tocilizumab to placebo, without background therapy, showed benefit in lung disease, but did not meet its primary skin outcome [10]. Tocilizumab is listed in the EULAR recommendations as an option for skin treatment for patients with early, inflammatory dcSSc, citing a trend toward benefit and an acceptable safety profile [11], though data are limited regarding combining tocilizumab with other therapies such as MMF. Our study provides clinical context to these guidelines. While many respondents have used tocilizumab for dcSSc skin in patients without ILD, it is uncommonly used as an early-line treatment for skin.

This study also provides real-world, contemporary context to patterns of referral for HSCT in dcSSc. Recent studies have generated new data suggesting that HSCT would not have shown a benefit if compared with contemporary standard-of-care instead of cyclophosphamide [18]. We show that while HSCT is still considered a dcSSc treatment option, only 6% of respondents refer any eligible patient based on the HSCT trials inclusion criteria, underscoring its current role as a later-stage approach in refractory disease.

Most respondents will use systemic corticosteroids for skin symptoms if needed, similar to prior studies. The decision to add prednisone for skin is influenced by RNA polymerase III status, given the association between steroid use, RNA polymerase III positivity, and scleroderma renal crisis [19–21]; 73% will prescribe prednisone in RNA polymerase III-negative-patients, but only 58% in RNA polymerase III-positive patients. Similarly, in 2012, 78% of SSc specialists would consider using steroids occasionally for skin symptoms, despite the risk of SRC [4]. In the 2018 SSc specialist survey, 65% would prescribe corticosteroids if needed [5]. Prior studies did not stratify by RNA Polymerase III status; thus, our study adds additional clinical nuance related to current practice patterns. Notably, we did not assess the use of corticosteroids for non-cutaneous manifestations, including musculoskeletal symptoms or cardiac involvement; therefore, these practice patterns should not be generalized beyond the management of inflammatory skin symptoms.

Almost half of SSc specialists indicated that they have or will make changes in the way they manage dcSSc skin involvement based upon the EULAR and/or BSR guidelines, which highlights the impact of these recent society recommendations. Previous iterations of the BSR and EULAR guidelines do appear to have impacted treatment decision-making for vascular complications of SSc [22]. It is uncertain whether the recommendations have caused SSc specialists to change treatment patterns, or if adding specific medications into guidelines has improved availability for treatments, particularly biologics. Regardless, our findings highlight the impact of the significant effort required to generate society guidelines which support the clinical practices of SSc specialists and general rheumatologists alike.

This study represents global perspectives for treating dcSSc skin involvement and provides timely context to recent SSc treatment guidelines. However, we acknowledge study limitations. The respondents represent a group of physicians with particular interest in SSc clinical trials, which may have influenced the reported preference for trial enrolment. Many physicians were members of more than one of the SSc specialist email distribution lists, which challenged the ability to accurately assess response rate. As an anonymous survey, comparisons between responders and non-responders could not be made, which limited assessment of non-response bias. Failure to deliver messages was not quantified, preventing any distinction to be made between failure to contact and non-response. The voluntary study design may result in voluntary-response bias, selecting for physicians with strong interest in skin treatment, which limits generalizability. Familiarity with recent guidelines was high in the respondents, which may not be generalizable to all SSc specialists. General rheumatologists, who represent a significant proportion of SSc care globally [23], may have differing responses and are not included in the present study, which includes a relatively small number of rheumatologists who care for patients with SSc. The sample size was also insufficient for statistical testing between subgroups, including geographical regions. The majority of respondents were from either North America or Europe, which limited assessment of global practice patterns in other regions.

In conclusion, our survey demonstrates MMF is most preferred as a first-line option for managing skin involvement in dcSSc. If MMF is not effective, most SSc specialists next prefer to refer patients to a clinical trial. At the same time, SSc specialists recognize increased biologic use (generally an exclusion criterion for trials) impacts clinical trial interest and eligibility of patients. These practice trends align with and are reflected in the recent EULAR and BSR guidelines. There is an ongoing need for drug development, especially in the treatment of early dcSSc, and this duality challenges trial design and recruitment. Optimal matching of patients to studies will be critical to develop and test novel disease-modifying agents in SSc in the modern treatment era.

## Supplementary Material

rkag086_Supplementary_Data

## Data Availability

The data underlying this article will be shared on reasonable request to the corresponding author.

## Notes

*Disclosure statement*: K.S.L. reports consulting: Boehringer Ingelheim; research support: Novartis. A.V. reports consulting: Jade Biosciences, Inc. J.D.P. reports speaker honoraria from Johnson & Johnson and Boehringer Ingelheim. J.D.P. has undertaken consultancy work for Johnson & Johnson, AbbVie, Celexor Bio, Liquidia, AstraZeneca, Boehringer Ingelheim, Sojournix Pharma, IsoMab pharma and Permeatus, Inc. J.D.P. has received educational support from CSL Vifor outside the submitted work. D.P.J. reports stock ownership in AstraZeneca and Cytodyn. R.D. reports consulting fees from Boehringer Ingelheim, Bristol-Myers-Squibb, AstraZeneca and Liquidia Technologies. R.F.S. reports receiving consulting fees from Roche-Genentech, GlaxoSmithKline, Sanofi, ChemoCentryx, Novartis, Galderma, Cytori, AstraZeneca, Amgen, AbbVie, Boehringer Ingelheim, Certa, Vera Therapeutics and Regeneron as well as grant support from GlaxoSmithKline, Roche-Genentech, AstraZeneca, Bristol Myers Squibb, Kadmon, Boehringer Ingelheim, Cytori, ChemoCentryx, Corbus Pharmaceuticals, Novartis, Amgen, AbbVie, Principia, Horizon and speaker honoraria from AstraZeneca. J.K.G. reports consulting fees from Boehringer Ingelheim, and Regeneron. The remaining authors have declared no conflicts of interest.
