# Supplementary material for: Treating skin involvement in diffuse cutaneous systemic sclerosis: results from an international scleroderma specialist survey
Source: Rheumatol Adv Pract. 2026 Jul 21;10(3):rkag086. doi: 10.1093/rap/rkag086 (PMC13424433; doi:10.1093/rap/rkag086)
Supplement: rkag086_Supplementary_Data [file rkag086_supplementary_data.docx]

**Supplementary Material**

1. **Supplemental Figure S1.** Duration of time systemic sclerosis specialists report using to determine first-line diffuse skin treatment efficacy
2. **Supplemental Figure S2**. Maximal dose of prednisone used to treat diffuse skin involvement in systemic sclerosis according to RNA polymerase III autoantibody status.
3. **Supplemental Figure S3**. Respondent-reported alignment of current diffuse skin treatment practice with British Society of Rheumatology (BSR) and European Alliance of Associations for Rheumatology (EULAR) guidelines. Results reflect answers from 94 respondents.
4. **Supplemental Table S1**. Characteristics of respondents who completed and did not complete the full survey.
5. **Supplemental Table S2**. Preferred approach to treating diffuse cutaneous systemic sclerosis (dcSSc) skin involvement for an individual without cardiopulmonary involvement who is planning pregnancy and taking either mycophenolate mofetil (MMF) or methotrexate (MTX).
6. **Supplemental Table S3**. Access to biologics for the treatment of diffuse skin involvement in systemic sclerosis, by region.
7. **Supplemental Table S4**. Summary of “Please specify” answers.
8. **Survey.** Treatment of Skin Involvement in Diffuse Cutaneous Systemic Sclerosis Survey

**
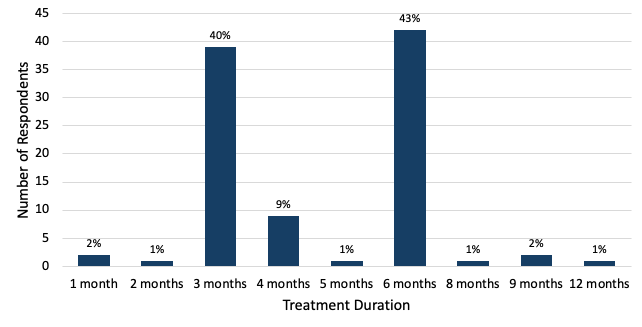
Supplemental Figure S1. Duration of time systemic sclerosis specialists report using to determine first-line diffuse skin treatment efficacy.**

**Supplemental Figure S2. Maximal dose of prednisone used to treat diffuse skin involvement in systemic sclerosis according to RNA polymerase III autoantibody status.**

**
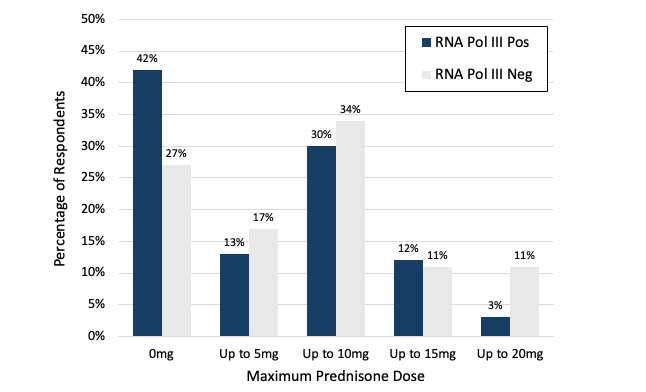
**

**Supplemental Figure S3. Respondent-reported alignment of current diffuse skin treatment practice with British Society of Rheumatology (BSR) and European Alliance of Associations for Rheumatology (EULAR) guidelines.** Results reflect answers from 94 respondents.


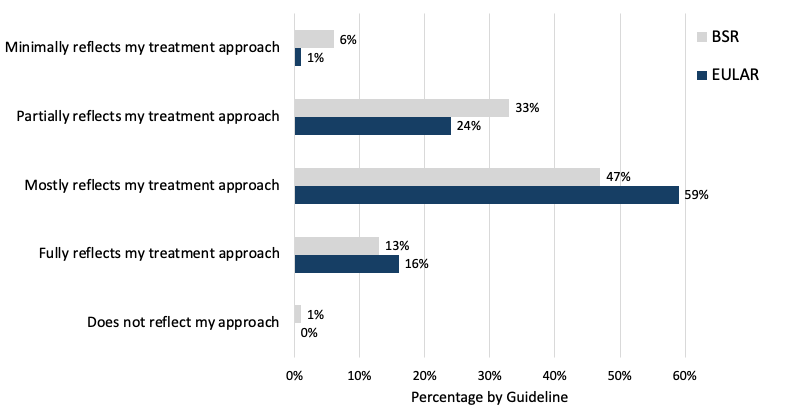


| **Supplemental Table S1. Characteristics of respondents who completed and did not complete the full survey** | | | |
| --- | --- | --- | --- |
| Factor | Incomplete  (N = 10) | Complete  (N = 93) | p-value |
| **Training level** |  |  | 0.52 |
| Fellow / Trainee | 1 (10%) | 3 (3%) |  |
| Faculty / Attending | 9 (90%) | 87 (94%) |  |
| Other | 0 (0%) | 3 (3%) |  |
| **Primary practice region** |  |  | 0.44 |
| North America | 5 (50%) | 39 (42%) |  |
| South America | 3 (30%) | 12 (13%) |  |
| Europe | 1 (10%) | 31 (33%) |  |
| Southeast Asia | 0 (0%) | 3 (3%) |  |
| East Asia | 0 (0%) | 1 (1%) |  |
| South Asia | 0 (0%) | 1 (1%) |  |
| Middle East and North Africa | 0 (0%) | 2 (2%) |  |
| Australia and Oceania | 1 (10%) | 4 (4%) |  |
| **Age** |  |  | 0.12 |
| 20-30 years old | 1 (10%) | 0 (0%) |  |
| 31-40 years old | 2 (20%) | 21 (23%) |  |
| 41-50 years old | 5 (50%) | 24 (26%) |  |
| 51-60 years old | 1 (10%) | 21 (23%) |  |
| 61-70 years old | 1 (10%) | 21 (23%) |  |
| 71-80 years old | 0 (0%) | 6 (6%) |  |
| **Sex** |  |  | 0.44 |
| Male | 2 (20%) | 37 (40%) |  |
| Female | 8 (80%) | 54 (58%) |  |
| I prefer not to answer | 0 (0%) | 2 (2%) |  |
| **Years in practice** |  |  | 0.32 |
| Currently still in training | 1 (10%) | 0 (0%) |  |
| 0-5 | 2 (20%) | 10 (11%) |  |
| 6-10 | 3 (30%) | 19 (20%) |  |
| 11-15 | 1 (10%) | 11 (12%) |  |
| 16-20 | 1 (10%) | 14 (15%) |  |
| 21-25 | 1 (10%) | 11 (12%) |  |
| 26-30 | 0 (0%) | 9 (10%) |  |
| >30 | 1 (10%) | 19 (20%) |  |
| **Primary practice setting** |  |  | 0.47 |
| Academic Center | 9 (90%) | 88 (95%) |  |
| Non-academic, clinical setting | 1 (10%) | 5 (5%) |  |
| **Primary practice location a Scleroderma Center** |  |  | 0.46 |
| No | 3 (30%) | 17 (18%) |  |
| Yes | 7 (70%) | 75 (81%) |  |
| Unsure | 0 (0%) | 1 (1%) |  |
| **Clinical Trial participation** |  |  | 0.12 |
| Principal Investigator | 5 (50%) | 66 (71%) |  |
| Co-investigator | 2 (20%) | 14 (15%) |  |
| Referred patients to a clinical trial only | 3 (30%) | 7 (8%) |  |
| No prior clinical trial experience | 0 (0%) | 6 (6%) |  |
| **Number of trials, among those who have participated in trials** |  |  | 0.54 |
| 1-5 | 7 (70%) | 41 (44%) |  |
| 6-10 | 0 (0%) | 17 (18%) |  |
| 10-15 | 1 (10%) | 15 (16%) |  |
| 15-20 | 1 (10%) | 9 (10%) |  |
| >20 | 1 (10%) | 11 (12%) |  |
| **Patient age range** |  |  | 0.59 |
| Adults only | 10 (100%) | 83 (89%) |  |
| Adults and children | 0 (0%) | 10 (11%) |  |
| **Monthly SSc patient volume (any subtype)** |  |  | 0.75 |
| 0-10 | 2 (20%) | 11 (12%) |  |
| 11-20 | 3 (30%) | 21 (23%) |  |
| 21-30 | 1 (10%) | 17 (18%) |  |
| 31-40 | 0 (0%) | 7 (8%) |  |
| 41-50 | 2 (20%) | 8 (9%) |  |
| 51-60 | 1 (10%) | 9 (10%) |  |
| >60 | 1 (10%) | 20 (22%) |  |

| **Supplemental Table S2. Preferred approach to treating diffuse cutaneous systemic sclerosis (dcSSc) skin involvement for an individual without cardiopulmonary involvement who is planning pregnancy and taking either mycophenolate mofetil (MMF) or methotrexate (MTX).** | |
| --- | --- |
| **Response** | **N = 96** |
| Switch MMF/MTX to azathioprine | 36 (38%) |
| Switch MMF/MTX to hydroxychloroquine | 2 (2%) |
| Switch MMF/MTX to IVIG | 7 (7%) |
| Switch MMF/MTX to rituximab | 14 (15%) |
| Switch MMF/MTX to a combination of other treatments | 6 (6%) |
| Stop MMF/MTX and monitor off treatment during pregnancy | 15 (16%) |
| Other | 8 (8%) |
| I do not recommend pregnancy for patients with dcSSc | 8 (8%) |

| **Supplemental Table S3. Access to biologics for the treatment of diffuse skin involvement in systemic sclerosis, by region.** | | | |
| --- | --- | --- | --- |
| **A. Rituximab Access** | | | |
|  | **No Access**  **(n = 0)** | **Yes, but limited**  **(n = 35)** | **Yes, without difficulty**  **(n = 68)** |
| North America | 0 (0%) | 14 (40%) | 30 (44%) |
| South America | 0 (0%) | 8 (23%) | 7 (10%) |
| Europe | 0 (0%) | 9 (26%) | 23 (34%) |
| Southeast Asia | 0 (0%) | 2 (6%) | 1 (1%) |
| East Asia | 0 (0%) | 0 (0%) | 1 (1%) |
| South Asia | 0 (0%) | 0 (0%) | 1 (1%) |
| Middle East, North Africa | 0 (0%) | 2 (6%) | 0 (0%) |
| Australia and Oceania | 0 (0%) | 0 (0%) | 5 (7%) |
| **B. Tocilizumab Access** | | | |
|  | **No Access**  **(n = 3)** | **Yes, but limited**  **(n = 35)** | **Yes, without difficulty**  **(n = 65)** |
| North America | 0 (0%) | 7 (20%) | 37 (57%) |
| South America | 2 (67%) | 11 (31%) | 2 (3%) |
| Europe | 1 (33%) | 9 (26%) | 22 (34%) |
| Southeast Asia | 0 (0%) | 2 (6%) | 1 (2%) |
| East Asia | 0 (0%) | 0 (0%) | 1 (2%) |
| South Asia | 0 (0%) | 0 (0%) | 1 (2%) |
| Middle East, North Africa | 0 (0%) | 2 (6%) | 0 (0%) |
| Australia and Oceania | 0 (0%) | 4 (11%) | 1 (2%) |
| Table reflects respondents’ answers to the question, “Do you have access to rituximab / tocilizumab (and/or biosimilar) for patients with systemic sclerosis where you practice?”, with the following answer options: (1) Yes, I can access this medication without difficulty, (2) Yes, I can access this medication, but access is limited/difficult, and (3) No. | | | |

| **Supplemental Table S4. Summary of “Please specify” answers.** | |
| --- | --- |
| **Please Specify Response** | **Count of Standardized Response** |
| **What is your current position/ level of training?** |  |
| Consultant | 2 |
| Professor | 1 |
| **Do you have access to rituximab (and/or biosimilar) for patients with systemic sclerosis where you practice?** |  |
| Insurance / approval | 29 |
| Cost | 4 |
| **Do you have access to tocilizumab (or biosimilar) for patients with systemic sclerosis where you practice?** |  |
| Insurance / approval | 21 |
| Cost | 12 |
| **In patients with dcSSc and mild and/or non-progressive ILD who have active skin disease despite mycophenolate, I would recommend as best next option:** |  |
| Add rituximab | 1 |
| Discuss all options | 1 |
| If inflammatory arthritis or TFRs present add tocilizumab; if not, refer for clinical trial | 1 |
| Refer to trial or add rituximab or tocilizumab if declines | 1 |
| **Which medication do you typically switch to or add? (After trying MTX, no ILD)** |  |
| MMF or rituximab | 1 |
| **Which medication do you typically switch to or add? (After trying something other than MMF or MTX, no ILD)** |  |
| Switch cyclophosphamide to MMF | 1 |
| **In patients who present with a new diagnosis of diffuse cutaneous systemic sclerosis (dcSSc) with active skin disease, but without ILD, my first line treatment for skin involvement is:** |  |
| Cyclophosphamide for high/rapid skin progression; MMF for moderate/slower skin progression. | 1 |
| **In patients who present with a new diagnosis of diffuse cutaneous systemic sclerosis (dcSSc) with active skin disease, and with mild and/or non-progressive ILD, my first line treatment for skin involvement is:** |  |
| MMF and discuss trials | 1 |
| **Assuming access to medication is without limitations, what would be your ideal induction treatment for a patient with early dcSSc, specifically who is Scl70 positive, with active skin and lung involvement?** |  |
| MMF + tocilizumab | 7 |
| MMF + rituximab | 4 |
| MMF + rituximab + nintedanib | 4 |
| MMF + tocilizumab or rituximab | 3 |
| MMF + nintedanib | 2 |
| MMF + low dose prednisone + nintedanib | 1 |
| CAR-T trial or stem cell transplant | 1 |
| CAR-T trial | 1 |
| Access it not without limitations | 1 |
| Nintedanib | 1 |
| No ideal treatment exists | 1 |
| **Assuming access to medication is without limitations, what would be your ideal induction treatment regimen for a patient with early dcSSc, specifically who is RNA Polymerase III positive, with active skin involvement?** |  |
| MMF + rituximab | 9 |
| MMF + tocilizumab | 3 |
| CAR-T trial | 1 |
| CAR-T trial or stem cell transplant | 1 |
| IVIG + MMF or MTX | 1 |
| MMF + biologics (unspecified), then stem cell transplant | 1 |
| MMF + MTX + IVIG | 1 |
| MMF + tocilizumab or rituximab or stem cell transplantation | 1 |
| MTX + rituximab or tocilizumab | 1 |
| No ideal treatment exists | 1 |
| **For patients with dcSSc who have active skin disease as well as myocarditis, my preferred treatment is:** |  |
| MMF + rituximab | 12 |
| MMF + tocilizumab | 6 |
| MMF + IVIG | 2 |
| Cyclophosphamide + MMF +/- low dose steroids or IVIG, clinical trial referral | 1 |
| Cyclophosphamide + pulse steroids | 1 |
| MMF + rituximab + low dose prednisone | 1 |
| MMF + low dose prednisone + IVIG | 1 |
| MMF + methotrexate + IVIG | 1 |
| MMF + prednisone + IVIG | 1 |
| MMF + rituximab + prednisone | 1 |
| MMF + rituximab and/or clinical trial | 1 |
| MMF + rituximab or IVIG | 1 |
| MMF + rituximab, possibly cyclophosphamide | 1 |
| MMF +/- rituximab | 1 |
| Rituximab + moderate-high dose steroids | 1 |
| Rituximab + moderate dose steroids | 1 |
| **Would you or have you prescribed rituximab (or biosimilar) for dcSSc skin involvement?** |  |
| Clinical trial population not representative of practice, insurance challenges | 1 |
| **Would you or have you prescribed tocilizumab (or biosimilar) for patients with dcSSc without interstitial lung disease?** |  |
| Inflammatory arthritis, skin | 1 |
| Inflammatory arthritis, skin, heart, high inflammatory markers | 1 |
| Inflammatory arthritis, tendon friction rubs, skin | 1 |
| Inflammatory arthritis, myocarditis | 1 |
| Myocarditis or in the presence of MMF side effects | 1 |
| Skin, tendon friction rubs, increased CRP | 1 |
| **Is there another disease manifestation that strongly influences your first-line treatment? Please specify.** |  |
| No | 13 |
| Tendon friction rubs | 5 |
| Non-SSc comorbidities | 3 |
| Skin progression rate | 3 |
| Cancer | 2 |
| Digital ulcers | 2 |
| Pulmonary hypertension | 2 |
| Disease duration | 2 |
| Disease duration, skin progression rate | 1 |
| Dysphagia, U1RNP positivity | 1 |
| GI symptoms | 1 |
| GI symptoms, weight loss | 1 |
| Interstitial lung disease | 1 |
| Lupus overlap, elevated IgG | 1 |
| Medication tolerance | 1 |
| Myositis or inflammatory arthritis | 1 |
| Digital vasculitis | 1 |
| Non-SSc comorbidities, age, infection risk | 1 |
| Non-SSc comorbidities, side effects, patient preferences | 1 |
| Raynaud's phenomenon | 1 |
| Scleroderma renal crisis | 1 |
| SSA positivity | 1 |
| Vasculitis | 1 |
| **What is your preferred approach to treatment of dcSSc significant skin disease for an individual without heart or lung involvement who is planning pregnancy and taking either methotrexate or mycophenolate?** |  |
| Avoid pregnancy if active | 1 |
| Avoid pregnancy if active, azathioprine if inactive | 1 |
| Avoid pregnancy if active, azathioprine or IVIG if inactive | 1 |
| Avoid pregnancy if active, if less active, azathioprine or rituximab | 1 |
| Azathioprine and hydroxychloroquine | 1 |
| Azathioprine or tocilizumab | 1 |
| Azathioprine with IVIG | 1 |
| Azathioprine, rituximab, or IVIG | 1 |
| Azathioprine, tacrolimus | 1 |
| Depends on clinical scenario | 1 |
| If controlled, tocilizumab or rituximab | 1 |
| Switch to rituximab | 1 |
| Tocilizumab | 1 |
| Tocilizumab and hydroxychloroquine | 1 |
| **For itch in patients with dcSSc, do you or would you prescribe any of the following?** |  |
| Emollients | 3 |
| Check for primary biliary cirrhosis, re: need for Ursodeoxycholic acid | 1 |
| Duloxetine | 1 |
| Dupixent or Xeljanz | 1 |
| Hydroxyzine | 1 |
| Leukotriene receptor antagonist | 1 |
| Montelukast | 1 |
| Montelukast, ondansetron | 1 |
| Topical gabapentin/menthol combination topical | 1 |
| **For telangiectasia in patients with dcSSc, do you or would you prescribe any of the following?** |  |
| Refer to dermatology | 4 |
| **In patients with dcSSc without ILD who have active skin disease despite mycophenolate, I would recommend as best next option:** |  |
| Add rituximab, work up for stem cell transplant, consider trial | 2 |
| Clinical trial or methotrexate | 1 |
| Clinical trial or rituximab | 1 |
| Clinical trial or tocilizumab | 1 |
| Dependent on patient preferences | 1 |
| **Would you enroll a patient with early, active diffuse cutaneous systemic sclerosis in a 12-month clinical trial that is placebo-controlled, without background therapy?** |  |
| If a patient is intolerant or declines treatment with MMF, MTX, rituximab | 1 |
| Yes, if patient prefers after discussion of alternative treatments | 1 |

**Treatment of Skin Involvement in Diffuse Cutaneous Systemic Sclerosis Survey**

Please note that the underlying branching logic of this instrument is not displayed below.

Treatment of Skin Involvement in Diffuse Cutaneous Systemic Sclerosis Survey

Please complete the survey below. Thank you!

Dear Colleague,

On behalf of the SCTC Skin Working Group, we invite you to complete this anonymous, online survey regarding the treatment of skin for patients with diffuse cutaneous systemic sclerosis. The purpose of this survey is to describe the current landscape of skin treatment practices for diffuse cutaneous systemic sclerosis, as of 2025.

This survey should take you approximately 10 minutes to complete.

By agreeing to complete and submit questionaries, you are consenting to participate in a research study. No identifying information will be recorded.

If you have questions about this study, please contact Kimberly Lakin, MD [(LakinK@hss.edu).](mailto:(LakinK@hss.edu) Thank you in advance for your participation,

Drs. Kimberly Lakin, Robert Spiera, Antonia Valenzuela, John Pauling and Jessica Gordon On behalf of the SCTC Skin Working Group

**I. Demographics**

What is your position/title? Physician


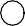

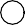

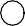

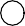

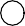

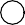


Nurse

Nurse Practitioner Physician's Assistant Research Assistant

Other, please specify:

What is your current position/ level of training? Fellow / Trainee Faculty / Attending

Other, please specify:


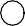

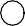

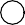


What is your primary region of practice? North America

Central America and the Caribbean South America


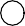

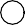

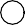

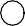

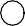

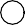

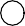

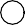

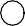

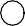

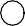

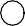


Europe Southeast Asia East Asia Central Asia South Asia

Middle East and North Africa Sub-Saharan Africa Australia and Oceania

Other, please specify:

What is your age range? 20-30 years old

31-40 years old


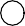

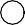

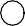

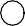

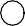

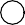

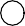


41-50 years old

51-60 years old

61-70 years old

71-80 years old

>80 years old

What sex were you assigned at birth? Male Female


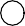

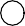

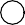


I prefer not to answer

How many years have you been in practice (i.e., years Currently still in training since training completed)? 0-5

6-10


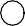

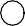

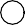

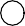

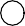

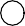

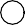

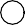


11-15

16-20

21-25

26-30

>30

How do you describe your primary practice setting ? Academic Center

Non-academic, clinical setting Other, please specify:


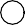

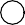

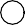


Is your primary location of practice a dedicated Yes

systemic sclerosis (SSc) center and/or clinic? No Unsure


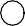

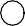

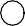


Does your primary location of practice have a program Yes for stem cell transplantation for SSc? No

Unsure


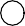

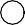

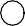


Have you participated as an investigator in clinical
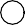
 Yes, I have participated as a principal investor trials in SSc? (including site-PI) and/or co-investigator

Yes, I have participated as a co-investor No, but I have referred patients to a clinical trial


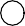

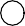


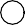
 No, and I have also not referred patients to a clinical trial

In approximately how many trials in SSc have you 1-5

participated, either as principal investigator or 6-10

co-investigator? 10-15

15-20

>20

What is the age range of patients you see with SSc? Adults only Children only Adults and children


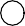

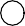

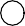

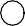

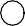

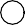

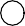

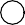


How many patients with SSc (any subtype) do you see in 0-10

a typical month? 11-20


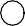

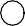

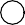

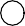

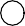

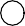

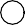


21-30

31-40

41-50

51-60

>60

Do you have access to rituximab (and/or biosimilar)
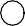
 Yes, I can access this medication without for patients with systemic sclerosis where you difficulty.

practice?
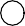
 Yes, I can access this medication, but access is

limited/difficult. Please specify why:


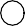
 No

Do you have access to tocilizumab (or biosimilar) for
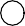
 Yes, I can access this medication without patients with systemic sclerosis where you practice? difficulty.


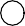
 Yes, I can access this medication, but access is limited/difficult. Please specify why:


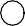
 No

**II. Treatment of diffuse cutaneous systemic sclerosis skin involvement**

In patients who present with a new diagnosis of Mycophenolate mofetil/mycophenolic acid diffuse cutaneous systemic sclerosis (dcSSc) with Methotrexate


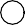

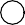

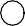

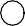

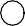

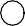

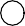

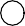

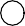

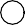

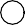

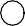

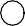

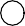

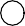

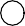


active skin disease, but without ILD, my first line Rituximab

treatment for skin involvement is: Tocilizumab Cyclophosphamide

Prednisone (low dose), monotherapy

Prednisone (moderate - high dose), monotherapy Intravenous Immunoglobulin Hydroxychloroquine

Azathioprine Abatacept Nintedanib

Stem cell transplant

Upfront combination therapy, please specify: Referral to clinical trial

Other, please specify:

In patients with dcSSc without ILD who have active
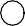
 Switch to another medication, but do not yet refer skin disease despite mycophenolate, I would recommend to clinical trial

as best next option:
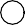
 Add another medication, but do not yet refer to clinical trial

Refer to clinical trial and continue mycophenolate Refer to clinical trial and switch to another agent Refer to clinical trial and add another agent


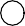

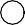

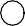

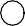

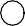


Refer for stem cell transplant evaluation Other, please specify:

Which medication do you typically switch to or add? Methotrexate Rituximab Tocilizumab Cyclophosphamide Prednisone (low dose)

Prednisone (moderate - high dose) Intravenous Immunoglobulin Hydroxychloroquine


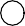


Azathioprine Abatacept Nintedanib

Stem cell transplant Referral to clinical trial

Other, please specify:

In patients with dcSSc without ILD who have active Switch to another medication, but do not yet refer skin disease despite methotrexate, I would recommend to clinical trial

as best next option: Add another medication, but do not yet refer to clinical trial

Refer to clinical trial and continue methotrexate Refer to clinical trial and switch to another agent Refer to clinical trial and add another agent Refer for stem cell transplant evaluation

Other, please specify:

Which medication do you typically switch to or add? Mycophenolate mofetil/mycophenolic acid

Rituximab Tocilizumab Cyclophosphamide Prednisone (low dose)

Prednisone (moderate - high dose) Intravenous Immunoglobulin Hydroxychloroquine

Azathioprine Abatacept Nintedanib

Stem cell transplant Referral to clinical trial

Other, please specify:

In patients with dcSSc without ILD who have active Switch to another medication, but do not yet refer skin disease despite my first line agent, I would to clinical trial

recommend as best next option: Add another medication, but do not yet refer to clinical trial

Refer to clinical trial and continue my first line agent

Refer to clinical trial and switch to another agent Refer to clinical trial and add another agent Refer for stem cell transplant evaluation

Other, please specify:

Which medication do you typically switch to or add? Mycophenolate mofetil/mycophenolic acid

Methotrexate Rituximab Tocilizumab Cyclophosphamide Prednisone (low dose)

Prednisone (moderate - high dose) Intravenous Immunoglobulin Hydroxychloroquine

Azathioprine Abatacept Nintedanib

Stem cell transplant Referral to clinical trial

Other, please specify:

In patients who present with a new diagnosis of Mycophenolate mofetil/mycophenolic acid diffuse cutaneous systemic sclerosis (dcSSc) with Methotrexate

active skin disease, and with mild and/or Rituximab

non-progressive ILD, my first line treatment for skin Tocilizumab

involvement is: Cyclophosphamide

Prednisone (low dose), monotherapy

Prednisone (moderate - high dose), monotherapy Intravenous Immunoglobulin Hydroxychloroquine

Azathioprine Abatacept Nintedanib

Stem cell transplant

Upfront combination therapy, please specify: Referral to clinical trial

Other, please specify:

In patients with dcSSc and mild and/or non-progressive Switch to another medication, but do not yet refer ILD who have active skin disease despite to clinical trial

mycophenolate, I would recommend as best next option: Add another medication, but do not yet refer to

clinical trial

Refer to clinical trial and continue mycophenolate Refer to clinical trial and switch to another agent Refer to clinical trial and add another agent

Refer for stem cell transplant evaluation Other, please specify:

Which medication do you typically switch to or add? Methotrexate Rituximab Tocilizumab Cyclophosphamide Prednisone (low dose)

Prednisone (moderate - high dose) Intravenous Immunoglobulin Hydroxychloroquine

Azathioprine Abatacept Nintedanib

Stem cell transplant

Other, please specify:

In patients with dcSSc and ILD who have active skin Switch to another medication, but do not yet refer disease despite methotrexate, I would recommend as to clinical trial

best next option: Add another medication, but do not yet refer to clinical trial

Refer to clinical trial and continue methotrexate Refer to clinical trial and switch to another agent Refer to clinical trial and add another agent Refer for stem cell transplant evaluation

Other, please specify:

Which medication do you typically switch to or add? Mycophenolate mofetil/mycophenolic acid

Rituximab Tocilizumab Cyclophosphamide Prednisone (low dose)

Prednisone (moderate - high dose) Intravenous Immunoglobulin Hydroxychloroquine

Azathioprine Abatacept Nintedanib

Stem cell transplant

Other, please specify:

In patients with dcSSc and ILD who have active skin Switch to another medication, but do not yet refer disease despite my first-line agent, I would recommend to clinical trial

as best next option: Add another medication, but do not yet refer to clinical trial

Refer to clinical trial and continue my first line agent

Refer to clinical trial and switch to another agent Refer to clinical trial and add another agent Refer for stem cell transplant evaluation

Other, please specify:

Which medication do you typically switch to or add? Mycophenolate mofetil/mycophenolic acid

Methotrexate Rituximab Tocilizumab Cyclophosphamide Prednisone (low dose)

Prednisone (moderate - high dose) Intravenous Immunoglobulin Hydroxychloroquine

Azathioprine Abatacept Nintedanib

Stem cell transplant

Other, please specify:

Assuming access to medication is without limitations, Mycophenolate mofetil/mycophenolic acid what would be your ideal induction treatment regimen Methotrexate

for a patient with early dcSSc, specifically who is Rituximab

RNA Polymerase III positive, with active skin Tocilizumab

involvement? Cyclophosphamide

Prednisone (low dose), monotherapy

Prednisone (moderate - high dose), monotherapy Intravenous Immunoglobulin Hydroxychloroquine

Azathioprine Abatacept Nintedanib

Stem cell transplant

Upfront combination therapy, please specify: Referral to clinical trial

Other, please specify:

Assuming access to medication is without limitations, Mycophenolate mofetil/mycophenolic acid what would be your ideal induction treatment for a Methotrexate

patient with early dcSSc, specifically who is Scl70 Rituximab

positive, with active skin and lung involvement? Tocilizumab Cyclophosphamide

Prednisone (low dose), monotherapy

Prednisone (moderate - high dose), monotherapy Intravenous Immunoglobulin Hydroxychloroquine

Azathioprine Abatacept Nintedanib

Stem cell transplant

Upfront combination therapy, please specify: Referral to clinical trial

Other, please specify:

For patients with dcSSc who have active skin disease Mycophenolate mofetil/mycophenolic acid as well as myocarditis, my preferred treatment is: Methotrexate

Rituximab Tocilizumab Cyclophosphamide

Prednisone (low dose), monotherapy

Prednisone (moderate - high dose), monotherapy Intravenous Immunoglobulin Hydroxychloroquine

Azathioprine Abatacept Nintedanib

Stem cell transplant

Upfront combination therapy, please specify: Referral to clinical trial

Other, please specify:

How long do you treat a patient before determining if 1 month your first-line medication for skin involvement is 2 months

effective? 3 months

1. months
2. months
3. months
4. months
5. months
6. months
7. months
8. months
9. months

Greater than 12 months

Would you or have you prescribed rituximab (or Yes

biosimilar) for dcSSc skin involvement? No, I do not think rituximab is helpful for skin involvement and/or additional data is needed

No, I do not have access to rituximab (or biosimilar) where I practice for patients with SSc No, for both of the reasons above

No, for another reason (please specify)

In recent years (i.e. within the last 2-3 years), my Increased

tendency to prescribe rituximab (or biosimilar) for Stayed the same

dcSSc skin involvement has: Decreased

I do not prescribe rituximab (or biosimilar) for dcSSc skin involvement

Would you or have you prescribed tocilizumab (or Yes, for refractory skin involvement biosimilar) for patients with dcSSc without Yes, but only if the patient has inflammatory

interstitial lung disease? arthritis

Yes, for another reason. Please specify: No I do not think that tocilizumab is helpful for skin and/or additional data is needed

No, I do not have access to tocilizumab (or biosimilar) where I practice for patients with SSc

No, for both of the reasons listed above

In recent years (i.e. within the last 2-3 years), my Increased tendency to prescribe tocilizumab (or biosimilar) for Stayed the same dcSSc skin involvement has: Decreased

I do not prescribe tocilizumab (or biosimilar) for dcSSc skin involvement

In recent years (i.e. within the last 2-3 years), my Increased

tendency to prescribe mycophenolate mofetil or Stayed the same

mycophenolic acid for dcSSc skin involvement has: Decreased

In recent years (i.e. within the last 2-3 years), my Increased

tendency to prescribe methotrexate for dcSSc skin Stayed the same

involvement has: Decreased

What is the maximum dose of prednisone (or equivalent) 0mg, I do not prescribe steroids for dcSSc that you would prescribe to a patient with dcSSc, RNA patients who are polymerase III positive polymerase III positive, for managing inflammatory Up to 5mg

skin symptoms: Up to 10mg

Up to 15mg Up to 20mg Up to 40mg Up to 60mg

Any dose, including pulse if needed

What is the maximum dose of prednisone (or equivalent) 0mg, I do not prescribe steroids for dcSSc you would prescribe to a patient with dcSSc, RNA patients who are polymerase III negative polymerase III negative, for managing inflammatory Up to 5mg

skin symptoms: Up to 10mg

Up to 15mg Up to 20mg Up to 40mg Up to 60mg

Any dose, including pulse if needed

What is the maximum dose of prednisone (or equivalent) 0mg, I do not prescribe steroids for dcSSc you would prescribe to a patient with dcSSc, RNA patients who are polymerase III positive polymerase III positive, for managing inflammatory Up to 5mg

joint and/or muscle symptoms: Up to 10mg Up to 15mg Up to 20mg Up to 40mg Up to 60mg

Any dose, including pulse if needed

What is the maximum dose of prednisone (or equivalent) 0mg, I do not prescribe steroids for dcSSc you would prescribe to a patient with dcSSc, RNA patients who are polymerase III negative polymerase III negative, for managing inflammatory Up to 5mg

joint and/or muscle symptoms: Up to 10mg Up to 15mg Up to 20mg Up to 40mg Up to 60mg

Any dose, including pulse if needed

**For each statement, rate the degree to which specific disease manifestations influence your**

**first-line treatment**

The presence of mild/stable interstitial lung disease (ILD)

The presence of severe/progressive ILD

The presence of inflammatory arthritis

The presence of myositis The presence of myocarditis

Elevated acute phase reactants RNA Polymerase III positivity Scl-70 positivity

Very strongly

influences

Somewhat influences Minimally influences Does not influence at

all

Is there another disease manifestation that strongly

influences your first-line treatment? Please specify.

Do you or would you refer patients with dcSSc for stem Yes, for any patients who meet criteria of the SCT cell transplant? clinical trials

Yes, for a patient who meets criteria of the SCT clinical trials with active disease and has failed other treatment options

No, I do not think SCT is more effective in SSc compared to other treatment options that are available

No, I do not have access to SCT where I practice No, I do not think the risks of SCT outweigh the benefits

Other, please specify:

What is your preferred approach to treatment of dcSSc Switch Methotrexate or Mycophenolate Mofetil to significant skin disease for an individual without azathioprine

heart or lung involvement who is planning pregnancy Switch Methotrexate or Mycophenolate Mofetil to and taking either methotrexate or mycophenolate? hydroxychloroquine

Switch Methotrexate or Mycophenolate Mofetil to IVIG

Switch Methotrexate or Mycophenolate Mofetil to rituximab

Switch Methotrexate or Mycophenolate Mofetil to a combination of other treatments. Please specify:

Stop Methotrexate or Mycophenolate Mofetil and monitor off treatment during pregnancy

Other, please specify:

I do not recommend pregnancy for patients with dcSSc

**For itch in patients with dcSSc, do you or would you prescribe any of the following?**

Yes No

Antihistamines Gabapentin or pregabalin Mirtazapine

Low dose naltrexone Oral steroids, low dose Topical steroids

(Optional) Other, please specify:

**For telangiectasia in patients with dcSSc, do you or would you prescribe any of the following?**

Yes No

Skin camouflage

Injected sclerosing agents

Thermocoagulation (e.g., pulsed dye laser or intense pulse light therapy)

(Optional) Other, please specify:

**III. Impact of Recent Guidelines**

Have you read (or have been presented with) the "The Yes, I have read this guideline and it was 2024 British Society for Rheumatology guideline for presented to me

management of systemic sclerosis"? Yes, I have read this guideline but it has not been presented to me

Yes, the guideline was presented to me but I have not read it

No, I have neither read nor been presented it

Have you read (or have been presented with) the "EULAR Yes, I have read this guideline and it was recommendations for the treatment of systemic presented to me

sclerosis: 2023 update"? Yes, I have read this guideline but it has not been presented to me

Yes, the guideline was presented to me but I have not read it

No, I have neither read nor been presented it

Table 1. Summary of BSR and EULAR skin treatment guidelines

Please rate the degree to which the BSR guideline Fully reflects my treatment approach reflects your current treatment approach for dcSSc Mostly reflects my treatment approach skin involvement? Partially reflects my treatment approach

Minimally reflects my treatment approach Does not reflect my treatment approach at all

Please rate the degree to which the EULAR guideline Fully reflects my treatment approach reflects your current treatment approach for dcSSc Mostly reflects my treatment approach skin involvement? Partially reflects my treatment approach

Minimally reflects my treatment approach Does not reflect my treatment approach at all

I have, or anticipate that I will, make changes to the Yes

way in which I treat diffuse cutaneous systemic No sclerosis considering the EULAR and/or BSR recent

guidelines.

**IV. Clinical Trials for Diffuse Cutaneous Systemic Sclerosis**

Would you enroll a patient with early, active diffuse Yes, for any patient with early, active diffuse cutaneous systemic sclerosis in a 12-month clinical systemic sclerosis.

trial that is placebo-controlled, without background Yes, but only for patients without any lung therapy? involvement.

Yes, but only in other specific situations. Please specify:

No, I would not enroll a patient with dcSSc in a placebo-controlled trial of 12-month duration that does not allow background therapy.

No, I do not refer patients to clinical trials.

Please rate your agreement with the following statements:

I would refer patients with dcSSc, with active ILD, to Strongly Agree trials up to 6 months that are placebo controlled Agree

without background therapy. Disagree

Strongly Disagree

I would refer patients with dcSSc, without active ILD, Strongly Agree to trials up to 6 months that are placebo controlled Agree

without background therapy in patients. Disagree

Strongly Disagree

I would refer patients with dcSSc to a clinical trial Strongly Agree without background therapy, if there is a clear escape Agree

strategy within the trial protocol. Disagree

Strongly Disagree

**Please indicate the degree to which you agree to the following statements:**

I feel comfortable enrolling patients in a trial with uniform background mycophenolate mofetil treatment, even if that means changing a patient from their current treatment.

Strongly Agree Agree Neutral Disagree Strongly

Disagree

I prefer trials that allow background therapy at the discretion of the treating physician.

The use/availability of biologics like rituximab and tocilizumab have impacted my enrollment of patients in clinical trials for dcSSc.

In general, for patients without ILD who fail mycophenolate and/or methotrexate, and have active skin disease, I would prefer to prescribe rituximab rather than refer to a clinical trial.

In general, for patients without ILD who fail mycophenolate and/or methotrexate, and have active skin disease, I would prefer to prescribe tocilizumab rather than refer to a clinical trial.

In general, for patients with ILD who fail mycophenolate and/or methotrexate, and have active skin disease, I would prefer to prescribe rituximab rather than refer to a clinical trial.

In general, for patients with ILD who fail mycophenolate and/or methotrexate, and have active skin disease, I would prefer to prescribe tocilizumab rather than refer to a clinical trial.

Performing skin biopsies of the forearm for clinical trials is a barrier for patients to enroll in clinical trials.

There are current treatment options that work well for skin in dcSSc and should be tried before enrollment in a clinical trial.
